# Large and stable: actin aster networks formed *via* entropic forces

**DOI:** 10.3389/fchem.2022.899478

**Published:** 2022-08-25

**Authors:** Friedrich Fabian Spukti, Jörg Schnauß

**Affiliations:** ^1^ Peter Debye Institute for Soft Matter Physics, University of Leipzig, Leipzig, Germany; ^2^ Fraunhofer Institute for Cell Therapy and Immunology, Leipzig, Germany; ^3^ Unconventional Computing Laboratory, Department of Computer Science, University of the West of England, Bristol, United Kingdom

**Keywords:** actin, molecular crowding, entropic forces, biocomputing, network formation, biopolymer stability

## Abstract

Biopolymer networks play a major role as part of the cytoskeleton. They provide stable structures and act as a medium for signal transport. These features encourage the application of such networks as organic computation devices. While research on this topic is not advanced yet, previous results are very promising. The protein actin in particular appears advantageous. It can be arranged to various stable structures and transmit several signals. In this study aster shaped networks were self-assembled *via* entropic forces by the crowding agent methyl cellulose. These networks are characterised by a regular and uniquely thick bundle structure, but have so far only been accounted in droplets of 100 μm diameter. We report now regular asters in an area of a few mm^2^ that could be observed even after months. Such stability outside of an organism is striking and underlines the great potential actin aster networks display.

## 1 Introduction

The idea of biocomputing has arisen in the 20th century, but has increasingly become more interesting over the past years. The finite nature of rare earths, the increasing demand for computation and the development of artificial intelligence ask for new ways of computation. In particular, recyclable materials and parallel computing are of special interest. Organic proteins could possibly provide both as they are compostable and have the capability to form regular networks that show great theoretic potential (Huber et al., 2015; Siccardi and Adamatzky, 2016; Siccardi et al., 2016; Adamatzky, 2017; Adamatzky, 2019). Information can be transported through those networks in various ways, such as currents or solitons. Theoretic models suggest that logical gates could be implemented in protein (actin) bundle networks with excitation waves (Adamatzky et al., 2019a; Adamatzky et al., 2019b; Siccardi et al., 2020). Structural as well as frequency and activity based gates were presented to be implementable in this way (Adamatzky et al., 2019a).

In order to provide protein networks for *in vitro* experiments based on these theoretical considerations, key factors are their transport capacity, stability, regularity and scale. The protein actin appears to have a big potential in all these aspects. Being part of the cytoskeleton, its ability to polymerise, self-assembly, self-heal, and form various bundle structures make the protein a very powerful tool.

In contrast to other proteins, actin filaments are conductive to (ionic) currents, mechanical as well as voltage solitons, and travelling localisations (Tuszyński et al., 1995; Luchko et al., 2004; Tuszyński et al., 2004; Tuszyński et al., 2005a; Tuszyński et al., 2005b; Priel et al., 2006; Satarić et al., 2009; Satarić and Satarić, 2011; Kavitha et al., 2017).

While these features are basic for calculational purposes, the great potential lies not in single filaments but bundle networks. There are multiple mechanisms to create such bundle structures *in vitro* using counter-ion condensation (Huber et al., 2012), cross-linking (Nedelec et al., 1997; Surrey et al., 2001; Backouche et al., 2006; Smith et al., 2007; Huber et al., 2013), confinements (Soares e Silva et al., 2011; Deshpande and Pfohl, 2012; Alvarado et al., 2014) and crowding effects (Madden and Herzfeld, 1993; Hosek and Tang, 2004; Huber et al., 2013; Huber et al., 2015). Depending on the mechanism and used concentration, different structures are forming (Huber et al., 2015). Of particular interest for computational purposes are aster-shaped networks, which are regularly spaced star-like formations. It was shown that an aster network formed with the crowding agent methyl cellulose has a uniquely thick and strong bundle architecture (Huber et al., 2015; Schnauß et al., 2016a). This makes such networks especially promising to be used as organic computational basis.

The underlying principle was presented in detail in (Asakura and Oosawa, 1958; Huber et al., 2013; Huber et al., 2015; Schnauß et al., 2016a; Schnauß et al., 2016b; Glaser et al., 2016). Summarizing these findings, if molecular crowding agents at a sufficient concentration are present in an isotropic distribution of actin filaments, entropic forces are exerted onto the polymers. Arising depletion forces cause a regular bundle network formation due to energy minimisation. Hence, this process is also called self-assembly. Reassembly after deformation has been initially demonstrated on the mesoscopic level and is assumed likely also on the mm-scale (Schnauß, 2015).

Another advantage of the self-assembly of actin induced by an inert crowding agent is that no additional conductive properties are added to the system. In contrast, if counter-ions induce self-assembly, additional undesired ionic currents have to be taken into account. We created actin aster networks by triggering the formation *via* diffusion of methyl cellulose into the actin filament systems. So far asters were reported to remain unchanged for 24 h after evaporation in droplets of maximally 100 μm diameter (Huber et al., 2015). We are now able to consistently create surprisingly large aster networks with a diameter of several mm^2^, which remained unchanged even after 5 months. Analysing characteristics of the network structure with respect to stability, regularity and scale, we give deeper insight into these fundamental material properties. Thus, our discoveries underline the great potential methyl cellulose induced actin aster networks demonstrate for organic computational purposes.

## 2 Materials and methods

### 2.1 Protein preparation

Except for the proteins used, all chemicals were purchased from Sigma-Aldrich (St. Louis, United States). Actin was prepared from rabbit muscle as described previously (Gentry et al., 2009). The protein was stored in G-buffer (Tris-HCl 5 mM (pH 7.8), CaCl_2_ 0.1 mM, ATP 0.2 mM, DTT 1 mM (dithiothreitol), and 0.01% NaN_3_) at a concentration of 24.9 μM. Actin was rhodamine-phalloidin labelled, with 10% 10x-F-Buffer (Tris-HCl (pH 7.8) 100 mM, KCl 1.5 M, ATP 5 mM, MgCl_2_ 5 mM, CaCl_2_ 2 mM) and 1% RhPh at concentration 5 μM. This F-rhodamine-phalloidin-actin was kept in the dark at room temperature for 1 h to allow polymerisation. A mixture was prepared to inhibit photobleaching (Tris 50 mM (pH 7.8), MgCl_2_ 24 mM, ATP 12 mM, DTT 40 mM, Dabco 8.8 mM). BSA (bovine serum albumin) at 1% was then added to the mixture to prevent unspecific binding as it was reported to have no specific interactions with actin. Unlabelled and labelled actin were added quickly one after another in a ratio of 43.1% unlabelled actin, 24.0% labelled actin, 16.7% mixture and 16.2% BSA and then pipetted on a glass cover slip. Until depositing the final droplets all solutions apart from labelled actin were kept on ice.

### 2.2 Droplet deposition and diffusion

**FIGURE 1:**
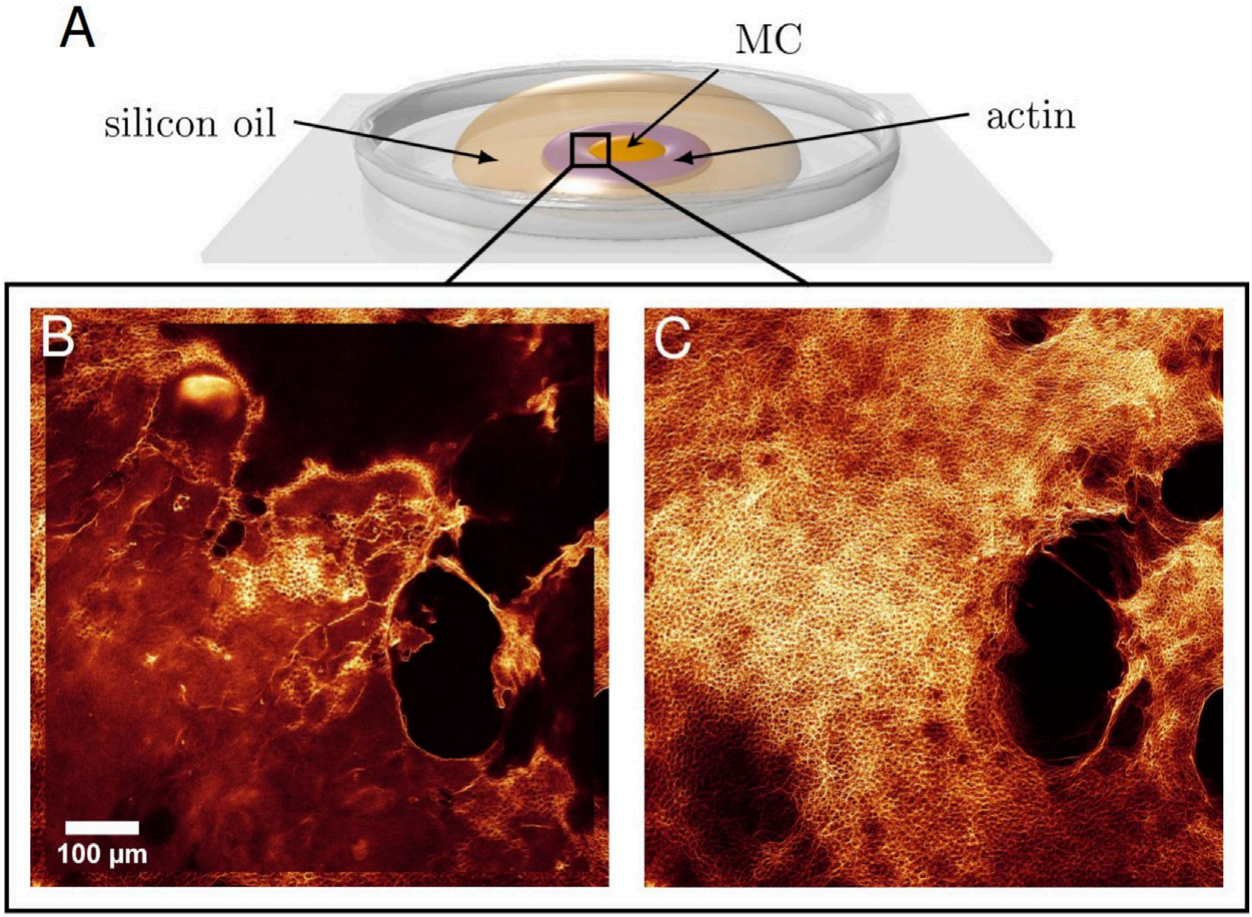
**(A)** Droplet Deposition. Directly after initializing the polymerisation of actin filaments, the solution was carefully pipetted around a droplet of MC. A silicon oil layer on top prevented evaporation. Diffusion of actin filaments and MC built up a concentration gradient. Thus, polymerisation and bundling were temporally separated. An isotropic polymer distribution is necessary for asters to form. **(B)** Formation of the aster networks ∼ 1 hour after the start of the experiment and **(C)** 4 hours later illustrate the time evolution of the system until reaching the final state.

A glass cover slip was prepared as portrayed in Figure 1 as an open setup. Two-component glue was used to prevent overflow. For aggregation by molecular crowding, MC (methyl cellulose) at 2% was deposited onto the cover slip with a droplet diameter of about 5 mm. The previously described solution with labelled and unlabelled actin was then carefully pipetted around the droplet in the form of a ring of width 2.5–5 mm (actin concentration 11.9 μM). Subsequently, the whole droplet was quickly covered by silicon oil (500cst) to prevent evaporation. Polymerisation of the unlabelled actin solution was immediately induced by adding KCl and MgCl_2_, to final concentrations of 36.1 and 4.1 mM, respectively. A concentration gradient at the former boundary between actin and MC formed by diffusion, which took a few hours. Thus, the direct polymerisation and diffusion-triggered bundling were temporally separated, which is the necessary condition to establish aster-like actin structures (Huber et al., 2015). Careful pipetting and slow diffusion reduced flow in order to establish an isotropic polymer distribution, which is vital for aster shaped networks to self-assemble through entropic depletion forces in the crowded MC environment (Huber et al., 2015). While asters were usually visible already after about 1 hour, they could still change considerably until settling in their final state (see Figure 1).

Using the method described, we were able to create aster shaped actin networks consistently. The samples were then transferred to a confocal laser scanning microscope (TCS SP2 AOBS; Leica Microsystems). To prevent photobleaching, observation time in the microscope was kept to a minimum and the samples were subsequently stored in dark.

### 2.3 Automated vertex detection and structure analysis

The experimentally observed actin aster networks were analysed using image analysis routines. The network structure was treated as 2-dimensional. To detect aster center positions, an automated software was written using *Matlab* (the Mathworks, R2020a).

First, network knots were identified using the fact that many bundles point towards these knots. This was achieved by applying a correlation analysis based on thin lines along different angles (here: 15 steps from 0 to π). The lines were elongated further to pronounce the network vertexes after overlaying the images derived for all directions. Second, blurring followed by threshold and erosion steps finally allowed us to discriminate the individual vertexes. When using images of good quality, the developed routine allowed tracing vertex positions in a very consistent manner. A simple Delaunay triangulation was used to connect the detected vertex positions to visually illustrate the distances to nearest-neighbouring centres [see also (Huber et al., 2012)].

Using the detected center positions, it was possible to derive the average (mean) distance between the aster vertexes and the connected neighbour vertexes. For the latter, the median was chosen as this results in an integer (the chance of a median in between two integers for the number of vertexes is very low). Standard deviations for both values reflect the spread. Though the program works with different pixel sizes and magnifications, a pixel size of 2048 showed the best compromise in image quality and time consumption. The average density of vertexes was set to be the number of found asters divided by the total image area and then scaled to 1 mm^2^. Additionally, the homogeneity of the aster distribution was estimated using the built-in *Matlab* bootstrap function and a kernel density function.

## 3 Results

The formation of aster shaped networks has been shown in various ways [(Nedelec et al., 1997; Surrey et al., 2001; Backouche et al., 2006; Smith et al., 2007; Huber et al., 2012; Huber et al., 2013; Huber et al., 2015)]. In this study, however, we exclusively used the molecular crowding agent methyl cellulose (MC) for its resulting particular bundle structure. An open gradient setup was chosen, which allows access to the sample in contrast to a closed setup. When the labelled actin solution, globular actin, the polymerisation-inducing mixture and BSA were mixed together and deposited on the glass slide together with the MC as described in the methods section, the concentration gradient was built immediately through diffusion. Since the diffusion process is rather slow, the onset of the bundling can be considered to take place later than the filament polymerisation. The isotropic distribution of polymers and the absence of flow are a crucial requirement for aster shaped networks to form (Huber et al., 2015). However, the networks reported previously only reached diameters of up to 100 μm. We are now able to build much larger, long-living aster networks.

### 3.1 Size of the networks

**FIGURE 2:**
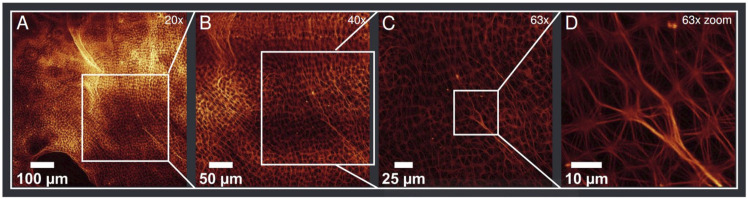
The same region of a sample with different objectives and additional zoom in **(D)**. Magnifications are given on the top right of each image **(A–D)**. **(A)** Shown is a large field of actin asters with preform of asters displayed at top and bottom of the image (see Supplementary Material). **(D)** Shows characteristic bundling of MC asters.

Typical sizes of a connected aster network structure range from 0.05 mm^2^ to a few mm^2^. The largest connected area was found to be more than 6 mm^2^. Beyond these pure aster fields, vastly bigger areas can be filled with preforms of asters that stem from a non-isotropic distribution or accidental flow in the solution. These forms have not been taken into account in the analysis, but show the potential to increase the spread of the aster area even more. Figure 2 shows a sample observed with objectives of different magnifications (from 20x to 63x). The figure displays both the bundling structure of MC asters and the area covered by asters in this sample. At the top and bottom of 2A “pre-aster” forms can be noted (see also Supplementary Material).

Interestingly, the expansion in *Z*-direction was found to be maximally 25 μm, but was usually lower around 10–15 μm. Comparing it to the distance between aster vertexes, which was around 10 μm, this means that the network had only one or two layers. In contrast to the large area spanned in *X*- and *Y*-direction, the network can be considered to be approximately 2-dimensional. This might be a favourable feature since it reduces complexity when the network is excited mechanically or electronically for computational purposes.

### 3.2 Stability

**FIGURE 3:**
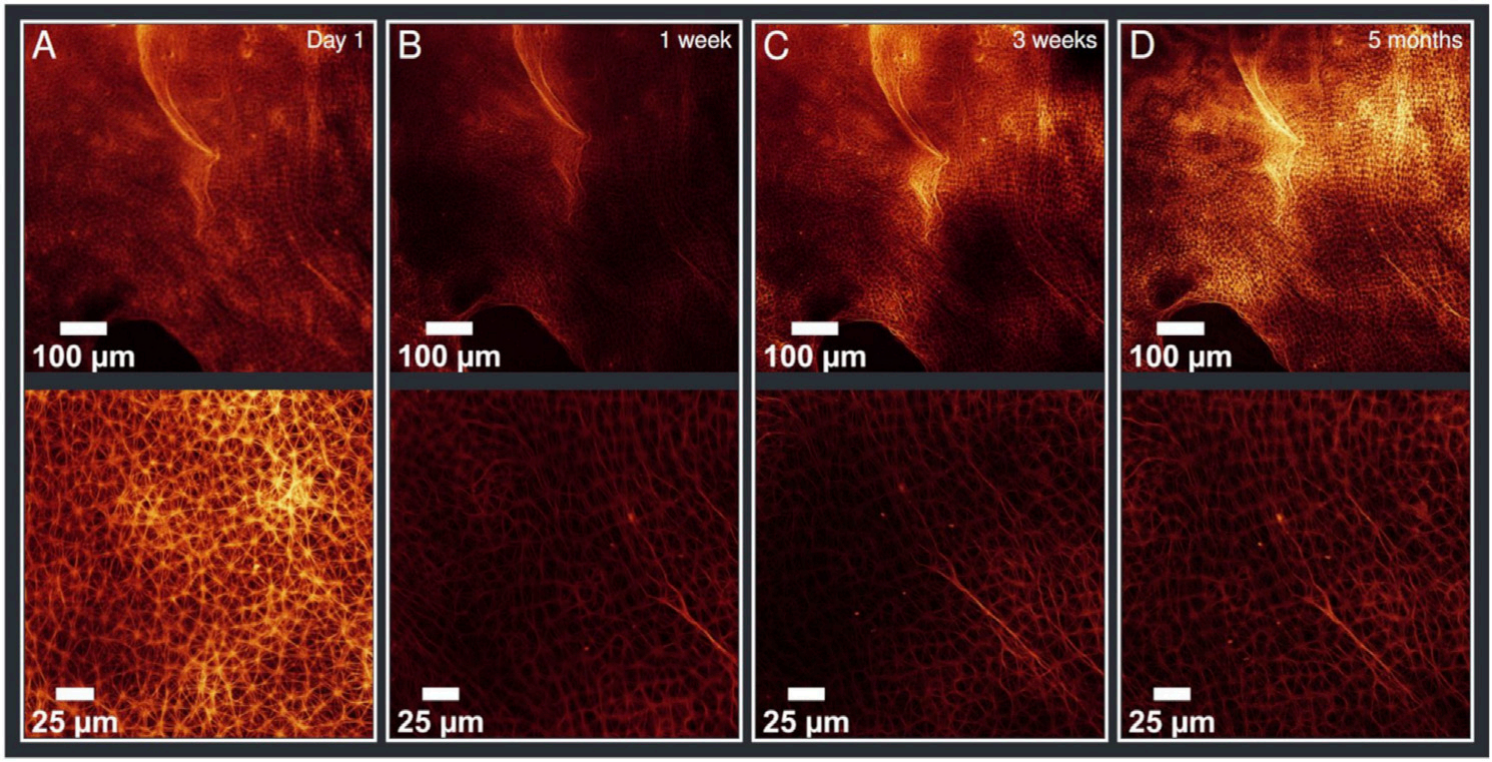
Evolution of the sample ordered by date with different magnifications (upper line ×20, lower line ×63 magnified). **(A)** taken on day 1 **(B)** after 1 week **(C)** after 3 weeks **(D)** after 5 months. There are no differences visible in this time evolution (on day 1 a different magnified section was imaged) demonstrating the stability of the aster network structure. According values of the analysis are given in Table 1.

After having reached their final structure, the aster networks were stable for multiple days. The structures were also detectable for weeks and even months. In fact, we were able to obtain images of a network after 5 months without changes in the network structure (Figure 3).

A quantitative evaluation described in the next section underlines the stability. Taking into account common deviations of the detection routine, the calculated values confirmed the visual expectation (Table 1). The average distances between the aster vertexes did not change over time. The median of connected neighbours was exclusively found to be 6. The vertex density shows deviations for different magnifications, which is addressed below. This issue, however, does not invalidate the assertion of an unchanged network structure over time.

**TABLE 1:** Image analysis was performed on the pictures in Figure 3. The results for each image are the average distance between aster vertexes (mean), connected neighbours (median), number of vertexes in 1 mm^2^ and correlation to the theoretical hexagon model.

Image	Av Dist	Neighb	Vert. Dens	Corr. Hex
Upper line (20x)				
Day 1	11.4	6	9,170	1.037
1 week	11.7	6	8,324	1.022
3 weeks	12.0	6	8,098	1.013
5 months	11.8	6	8,585	1.029
Lower line (63x)				
Day 1 magnified	11.9	6	8,200	1.004
1 week magnified	12.1	6	7,768	0.978
3 weeks magnified	11.9	6	7,768	0.975
5 months magnified	11.8	6	7,697	0.928

### 3.3 Aster recognition

**FIGURE 4:**
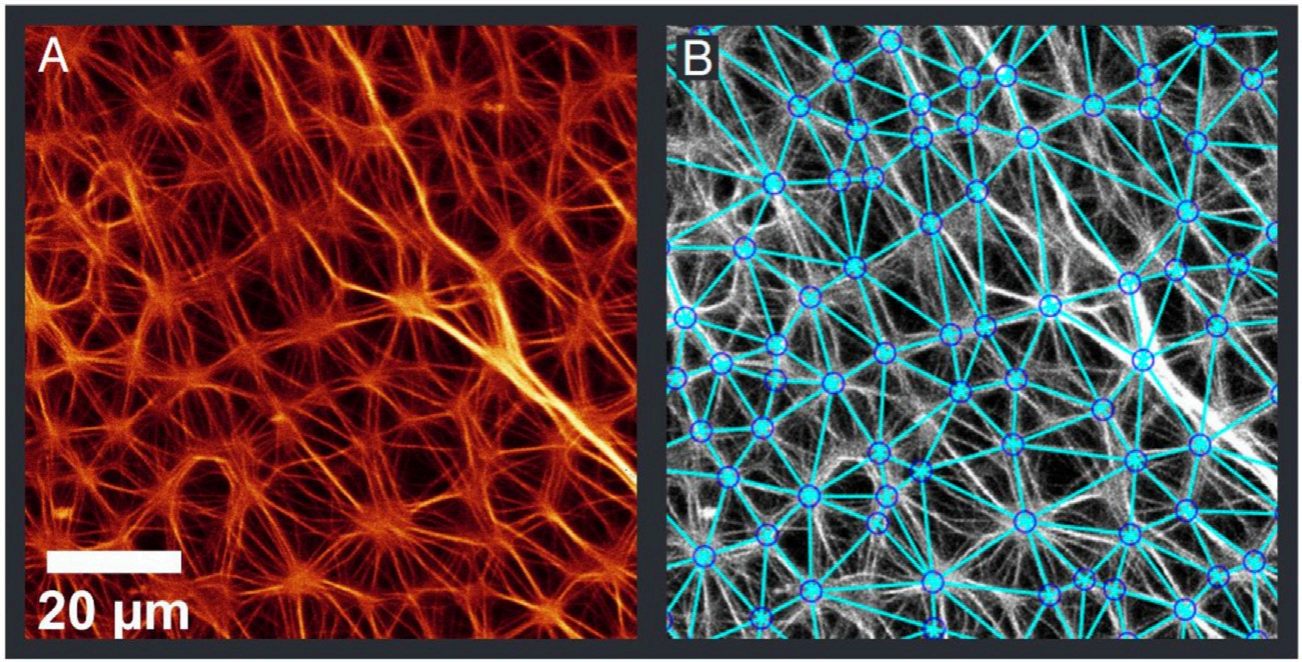
Original image **(A)** and detected aster vertexes **(B)**. The detection was performed with automated image analysis routines. The determined points were then connected with a Delaunay triangulation. Further analysis as seen in Figure 5 was done on the basis of the detected points.

**FIGURE 5:**
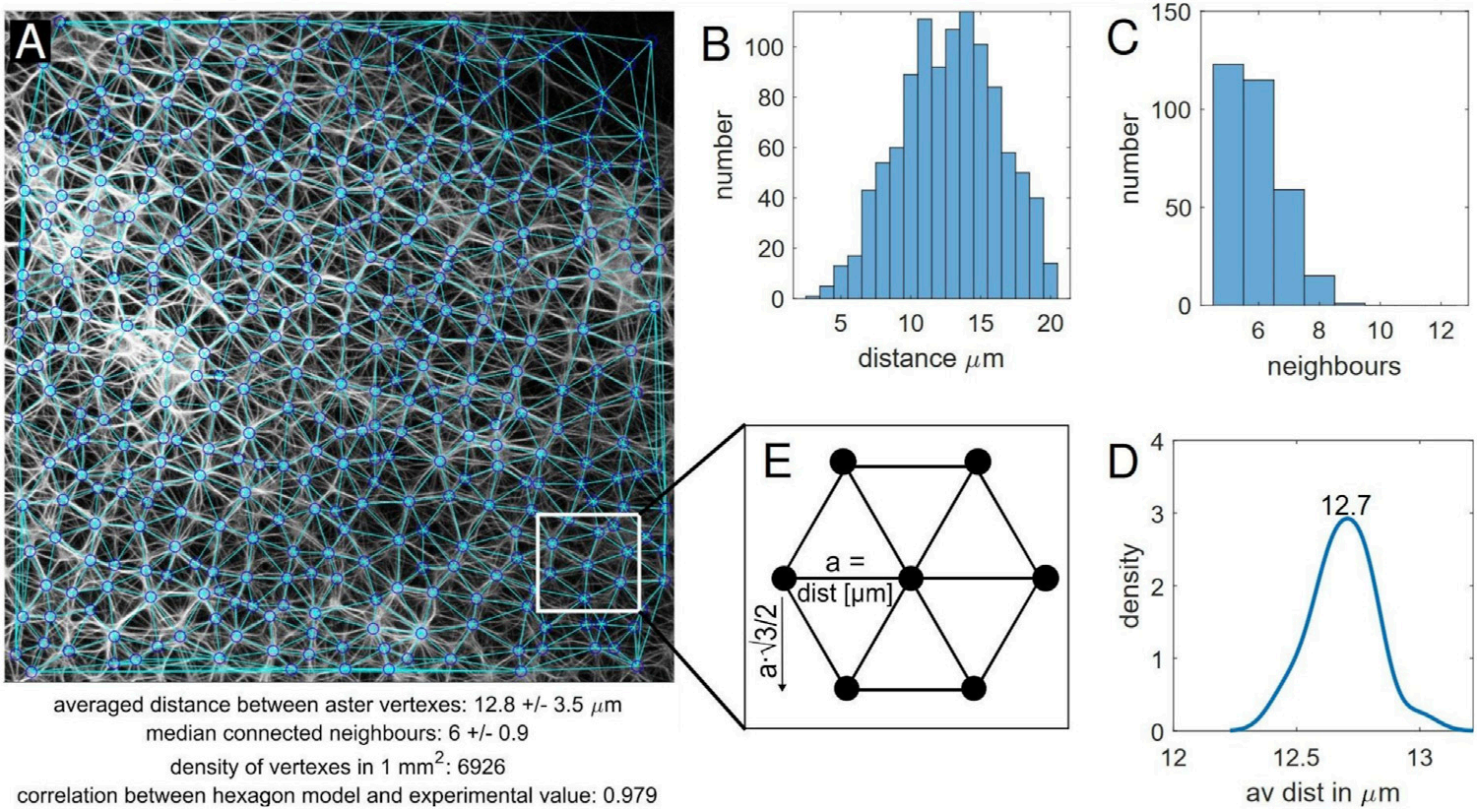
Aster vertexes recognition program. **(A)** The vertex detection and triangulation reflect the network, outcome values of the analysis are displayed at the bottom. Further shown are the **(B)** distribution of distances between the aster vertexes, **(C)** distribution of connected neighbour vertexes. **(D)** Bootstrapping was performed on the average distances between vertexes of different sections *via* the built-in Matlab function. A kernel density was determined out of the bootstrapped results. **(E)** Hexagon model, where the number of vertexes is calculated using the packing density.

A self-written *Matlab* program was used to analyse the fluorescence microscopy images in terms of network structure. The analysis included 120 images from 10 samples out of four independent preparations (see Supplementary Material for Matlab script and images). In the program, aster vertexes were detected and connected with a Delaunay triangulation, which reflects the actual network well (Figure 4). These data points were then used to calculate the average distance between the vertexes, the number of connected neighbours and the number of vertexes found in 1 mm^2^ (Figure 5A). The network structure was treated as 2-dimensional due to the minimal extension in *Z*-direction.

The distances between the vertexes within a sample usually vary from 3 to 20 μm (Figure 5B). Vertexes in a proximity of less than 3 μm were not considered to be individual vertexes, distances above 20 μm merely arose at the image edge or in parts of the image not covered with asters. Experimentally found values beyond these thresholds were not taken into account. The average distance between vertexes, however, ranged from 9 to 13 μm. While the standard deviation (typically ∼ 3.5 μm) reflects the spread of different distances, the mean value may still have an error of ± 0.5 μm as a result of image quality and the 2D image displaying in fact a 3D network.

The range of neighbouring vertexes in the triangulation ranged from 5 to 12 (Figure 5C). While values below five were only found at the image edge and were therefore ignored (negligible exceptions), 12 was the maximum value found out for all the samples. The highest occurrence was five and six neighbours. The median was hence almost exclusively found to be 6, with an interquartile range of 1. This finding also led to the comparison to a hexagon model, which is described in more detail below. Neighbouring vertexes often connect *via* multiple strands.

In order to check for homogeneity, analysis was performed with images of different magnifications (lowest displayed 750 × 750 μm). Variation for the average distance between vertexes was in the regime of 0.3 μm or less and the number of connected neighbours showed no significant difference. This means that the networks were fairly homogeneous. Hence, we presume that a sample does not have to be magnified a lot to be able to analyse the aster structures.

Furthermore, bootstrapping was performed to examine the homogeneity in more detail. For this purpose, an image was divided into 16 or 64 equal sections (depending on magnification) and then analysed as before with the *Matlab* program. The results for the connected neighbours were not adequate, as the sections were too small. The average distance between vertexes, however, showed only small variation indicating that indeed the aster network can be considered to be homogeneously distributed (Figure 5D).

Presuming a homogeneous distribution, we considered a certain regularity in the networks. A consequence should be a fairly constant density of vertexes in a given area. Accordingly, the number of vertexes found in 1 mm^2^ were counted and normalized with the size of the image in μm. Values were found between ∼ 6,800 and ∼ 10,500. Images obtained with lower magnification produced larger values. The reason for this was most likely that a larger area was imaged and hence there is comparably less influence of the image edges (where asters are not detected). In this aspect, the magnification plays a role.

Another aspect of the regular distribution is the arrangement of connected neighbours. As stated earlier, there is a high likeliness of six neighbours that are fairly well-arranged. In fact the recognition program reflected a lot of well-shaped polygons, in particular hexagons. This led to the idea to compare the asters to a perfectly regular equilateral hexagon structure. In the latter, vertexes were considered to be arranged in equilateral hexagons as shown in Figure 5E. The number of vertexes in 1 mm^2^ can then be found using the packing density of this structure, i.e. the distance between next neighbours in X- and *Y*-direction in a unit cell. In one direction the distance is exactly the “lattice constant” (in this case the experimentally determined average distance between vertexes), in the other $\sqrt{3}/2*$ this constant. With the following formula the number of vertexes in the hexagon model was then calculated, with *a* being the average distance between vertexes:

$$number\;of\;vertexes=\frac{1000\mu m}{a}*\frac{1000\mu m}{\frac{\sqrt{3}}{2}*a}$$

The two determined values for the number of vertexes were then compared. In most cases, the deviation was below 5%. There are, however, outliers that stem from bad image quality or sections of an image that were not covered with asters. Generally, images with a lower magnification revealed lower deviations between the experimental value and the model (∼ 1.5% more vertexes than in model for small magnification, ∼ 2.5% less than in the model for high magnification). From the results it can be concluded that the packing density of the hexagon model gives a good approximation of the number of vertexes found in a selected area given the average distance between the vertexes. The model hence could be used vice versa in order to estimate the average distance when the number of vertexes is known.

## 4 Discussion and outlook

*In-vitro* aster formation with the protein actin was thoroughly presented earlier. Actin aster networks have been shown to form with various methods (Nedelec et al., 1997; Surrey et al., 2001; Backouche et al., 2006; Smith et al., 2007; Huber et al., 2012; Huber et al., 2015). In particular, the self-assembly *via* crowding agents has been reported previously (Huber et al., 2015). We now used a different open system with the crowding agent methyl cellulose only. Advantages of our approach are the very easy lab routine to consistently create surprisingly large aster fields and accessibility to the sample as well as the long lasting structures. Our findings are to be considered an intermediate step towards a potential application of such networks for computational purposes. Presenting various characteristics of the network structure, we gave deeper insight into the fundamental material properties.

Methyl cellulose based networks in contrast to other crowding agents have a uniquely thick bundle structure. We found the average distance between the aster vertexes to be 9–13 μm. Despite individual bundles to be of different lengths, a regularity is still present in the pattern. *Via* bootstrapping we were able to show a homogeneous distribution over a large area. With images of different magnification this result could be additionally confirmed. Hence, it seems to be sufficient to analyse the structures the networks with lower magnifications, i.e. larger field of views.

The regularity is further reflected in the number of connected neighbouring vertexes with a strong tendency towards five to six neighbours. Accordingly, a lot of fairly equilateral polygons are reflected by the recognition program. A comparison to the hexagon packing density revealed that indeed the latter can be used as an approximation for the number of vertexes found in a given area with deviations of about 5%. It could be argued that the polygons are a feature of the triangulation. However, the pattern recognition shows large coincidence with the actual network and, more importantly, the number of vertexes is independent of the triangulation. Therefore, the correlation to the hexagon model is a proof of the regularity.

Taking a look at the whole network dimensions, we were able to create aster fields of previously unreported size reaching up to a few mm^2^ in size. Due to the consistency we presume that our new setup routine accounts for this. While the areas found are already unexpectedly large, there is potential to increase them even further. A crucial requirement remains, which is an isotropic distribution of polymers and the absence of flow. Apart from the scaling, another surprising result is the stability over months. Such a long stable structure for protein networks has not been reported yet. Our results therefore show very promising features that, to date, are only attributed to actin aster networks created by entropic forces.

It can be expected that the actin monomers denature within a few days and that the samples must have dried out after these long observation times. Future studies will be aimed to clarify when the denaturation occurs and if/when the actin bundles lose their information conducting properties. Upon loss of these properties, it may even be possible that the structures can serve as a template to reestablish conducting actin bundles in the same structure. It seems reasonable that the denatured structures can be used to grow new actin filaments, a procedure which might be enhanced by including nucleation proteins such as Arp2/3, gelsolin or N-Wasp, which exclusively bind to actin structures (Wear et al., 2000; Pollard, 2016). If according binding sites at the denatured actin structures are still usable or if new binding domains, for instance based on DNA-nanotechnology (Lorenz et al., 2018), need to be employed has to be clarified in future experiments. Further research will also be needed to investigate if the conducting properties are comparable after the structures have been reestablished. Since no reliable experiments have been established yet to compare and explain the conductivity of these comparably complex structures, we can only speculate about certain possibilities at this point.

The open setup, however, enables new experimental studies. Measuring devices can be inserted to excite the networks electronically and/or mechanically. This new approach allows to examine whether a different response happens in presence of an aster network and if it is possible to place an electrode onto a vertex to measure excitations within particular bundles.

Additionally, the large scale of these networks enables rheological studies to study the effect of deformations onto mechanical and conductive properties of these networks. Using rheological methods allows global deformations in high force regimes. The patterns are expected to be strongly deformed even beyond breakage of single bundles destroying the regular shape. Since the networks are formed by energy minimising entropic forces, they could possibly reassemble into their original structure (Schnauß, 2015). If this were true, actin asters would not only be very stable (mechanically and over time), but also incredibly robust.

Anyhow our experiments are a major step forward in using these aster-like networks for computing purposes. Theoretical studies have proven the high potential for implementations of logical functions (Huber et al., 2015; Siccardi and Adamatzky, 2016; Siccardi et al., 2016; Adamatzky, 2017; Adamatzky, 2019). Our setup enables a practical approach with the prospect of massive parallelisation of computing capacities. The dimension, regularity and stability of the networks further could be an excellent basis for a wide range of applications, which can now be readily tested with our new setups and findings.

## Data Availability

The datasets presented in this study can be found in online repositories. The names of the repository/repositories and accession number(s) can be found below: http://dx.doi.org/10.25532/OPARA-180.
