# Supplementary material for: Large and stable: actin aster networks formed via entropic forces
Source: Front Chem. 2022 Aug 25;10:899478. doi: 10.3389/fchem.2022.899478 (PMC9481034; doi:10.3389/fchem.2022.899478)
Supplement: Supplementary file 1 [file DataSheet1.zip › Supplementary Images.pdf]

Supplementary Material

Analyses presented in this publication were performed on images provided at:
http://dx.doi.org/10.25532/OPARA-180

Additional images on preforms of asters, asters from different independent samples and illustrations of the extension in z-direction are shown within this document. To illustrate the 3D extension in the z-direction, videos were attached as additional supplementary material. The names of the mentioned samples refer to the images and folder structure in the repository mentioned above.

1 PREFORMS OF ASTERS

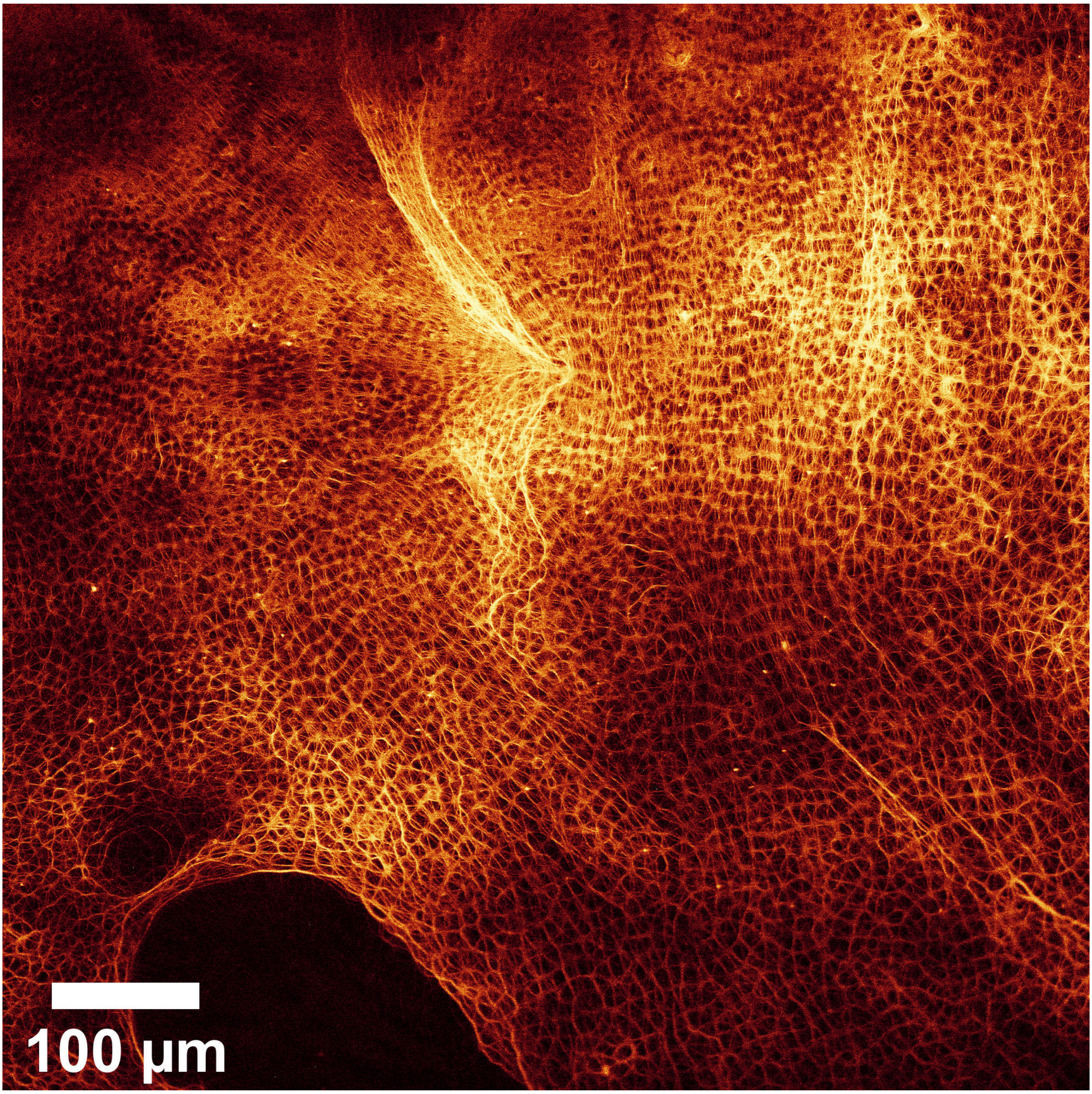

Figure S1. This image is also shown in Fig. 2A of the main manuscript. Preforms of asters are visible in the top left corner next to the bright region, where we assume that flow was present during the formation process destroying the isotropic distribution of filaments. Preforms of asters are also visible at the bottom of the image on the right side of the dark area where bundles display different characteristics.

2 IMAGES FROM DIFFERENT PREPARATIONS

Images presented in the main manuscript stem from one independent sample (Sample 1-1, prep. 1, glass slide 1) for illustration purposes. Fig. 1 is an exception and the image was taken from the fourth independent sample (Sample 4-2). In the following, images from other independent samples are shown (Figs. S2 - S4).

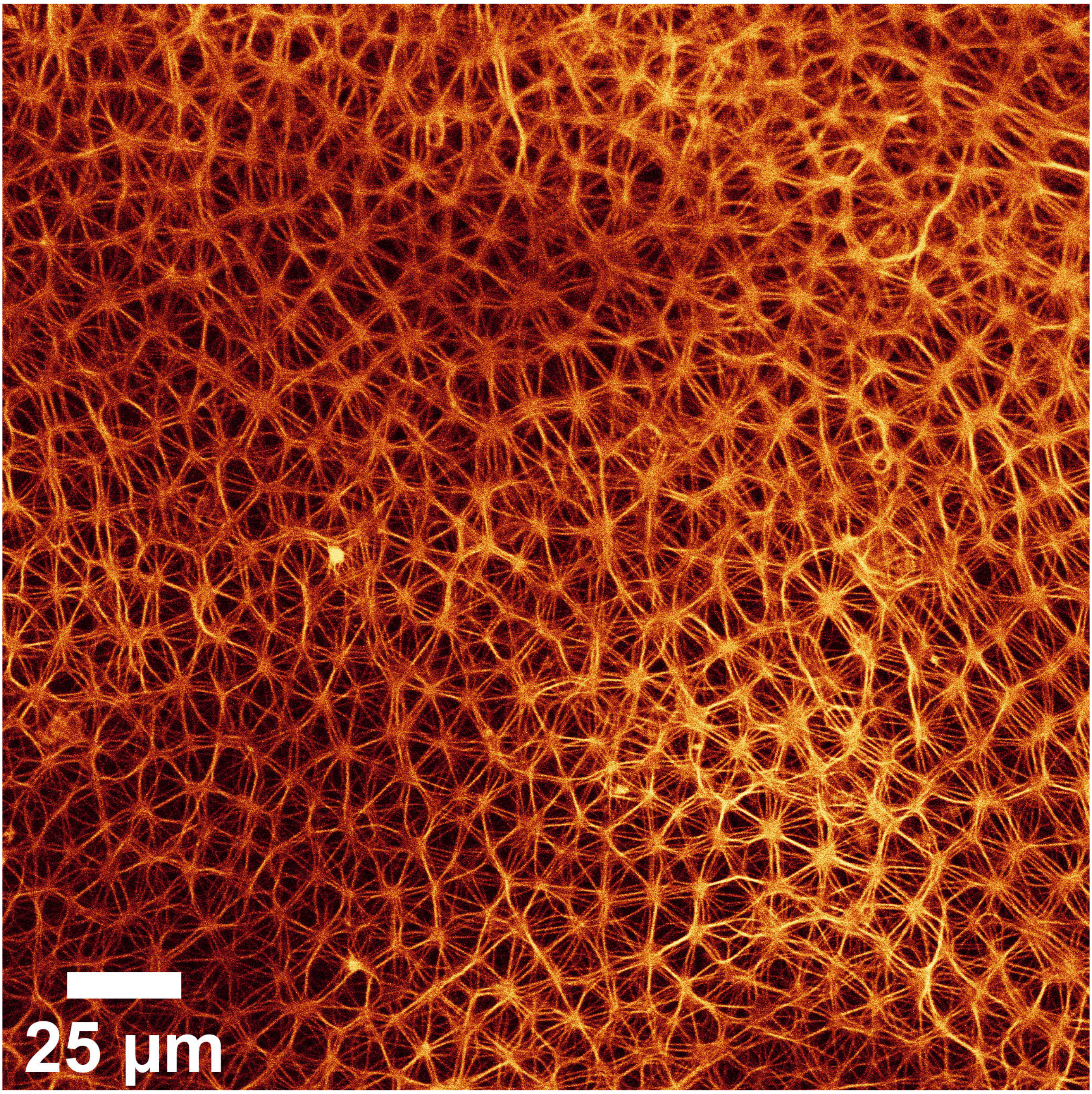

Figure S2. Preparation 2 (Sample 2-2)

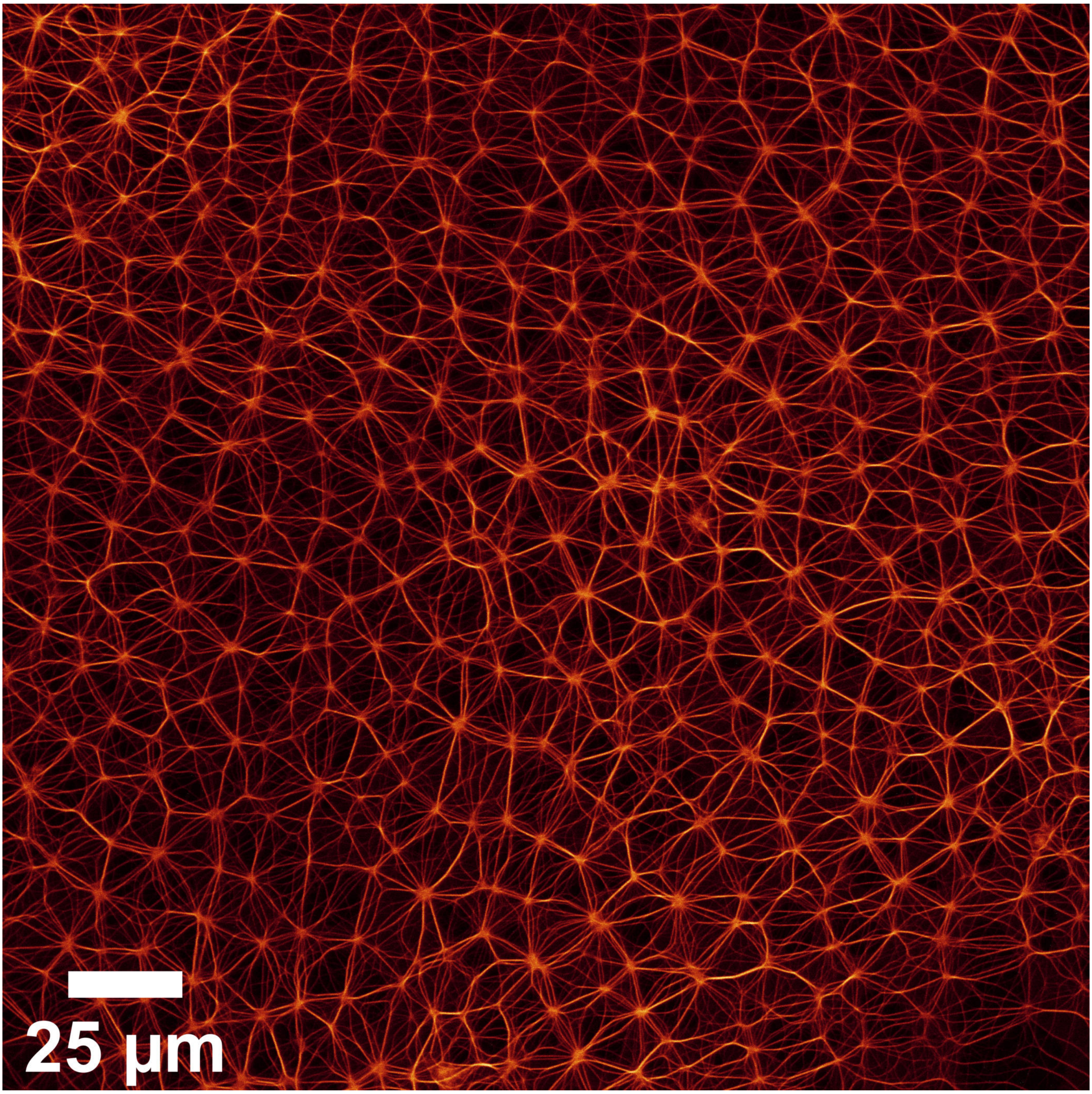

Figure S3. Preparation 3 (Sample 3-1)

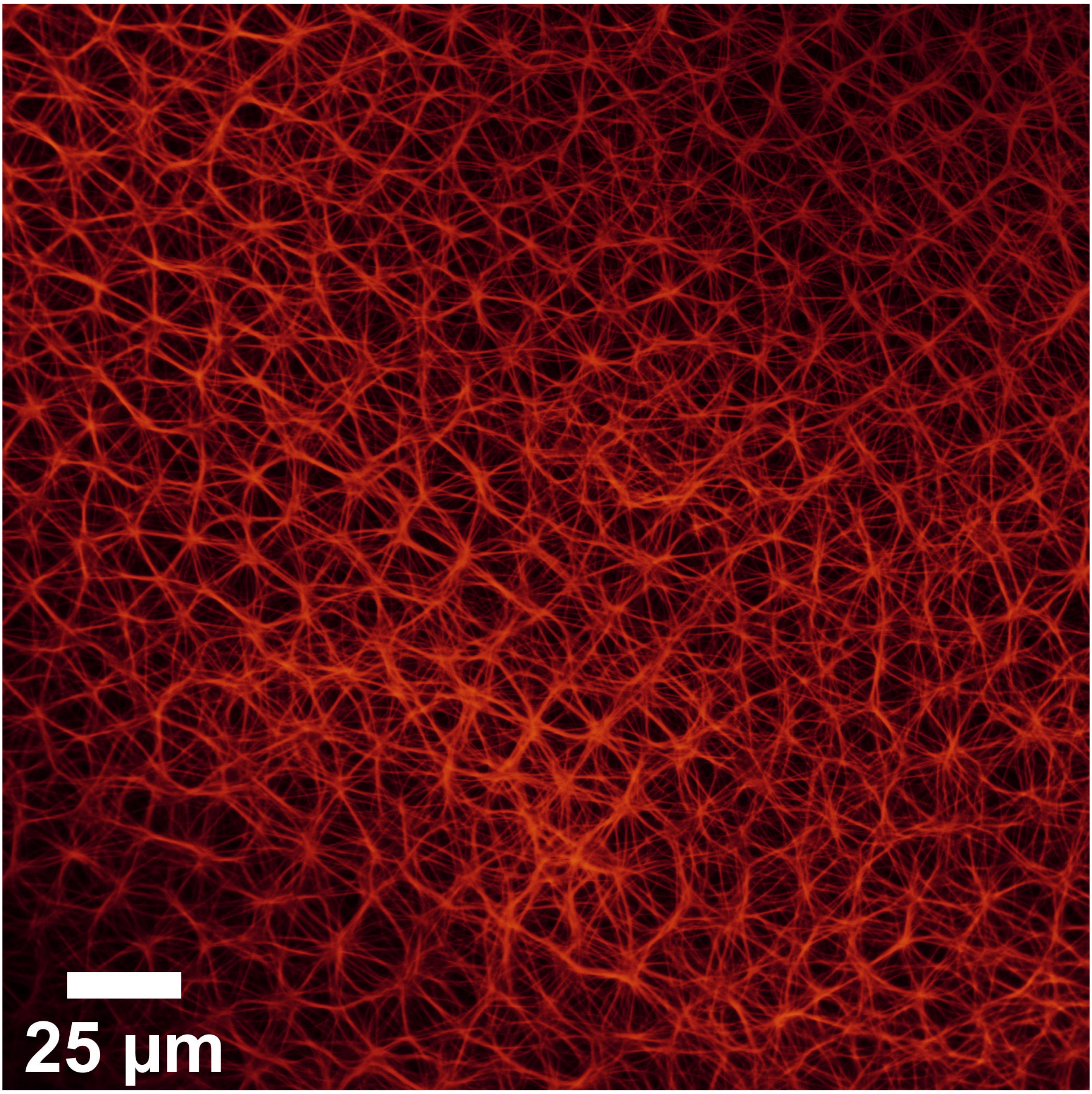

Figure S4. Preparation 4 (Sample 4-1)

3 STACKED IMAGES

Image stacks showing the extension in z-direction are presented below (Figs. S5 - S9). These images were taken from three different samples. According videos are also provided as supplementary material and can be identified by the naming scheme.

3.1 Sample 2-2

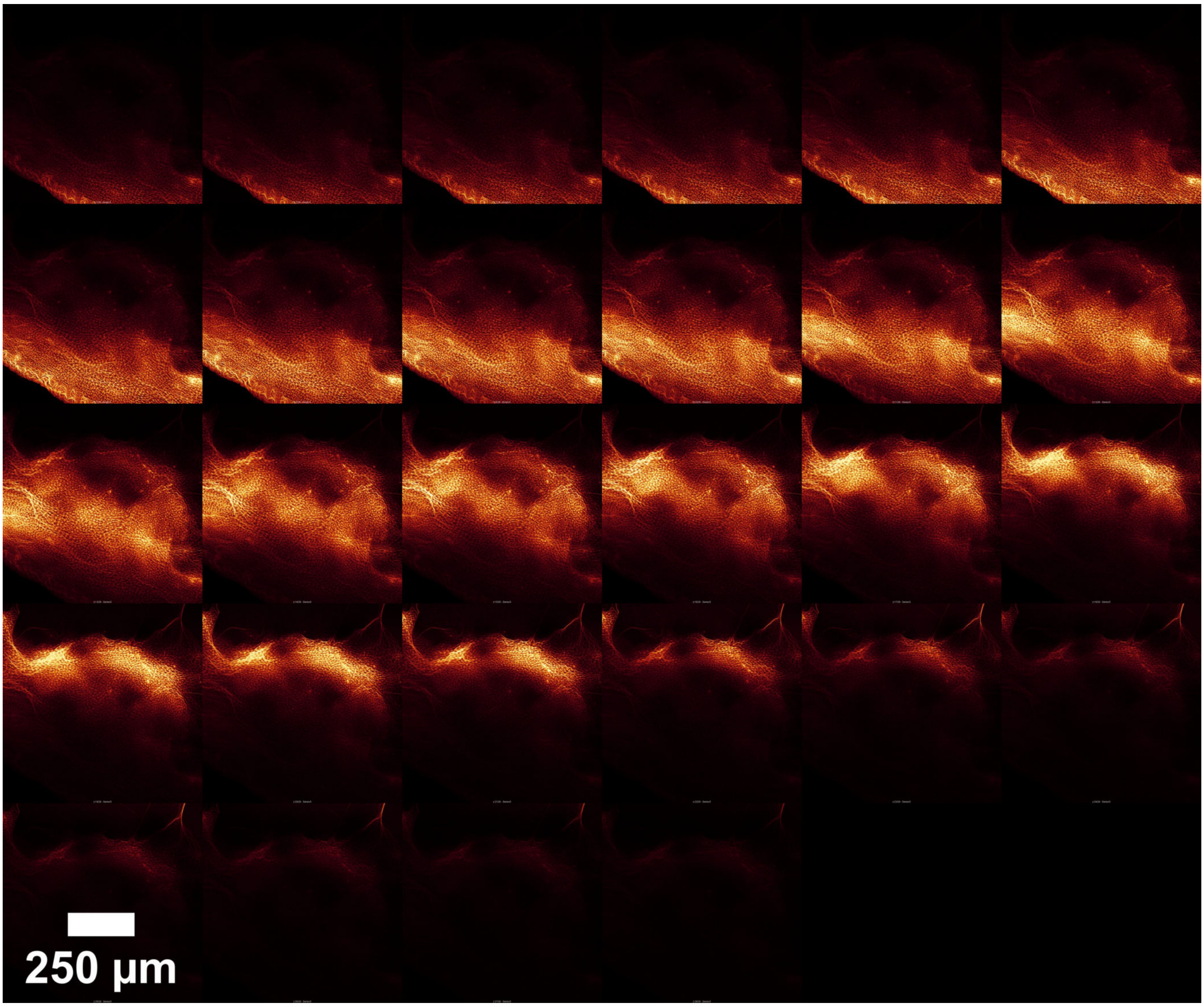

Figure S5. Sample 2-2 Montage. Images are ordered row-wise from left to right. An additional video is attached.

3.2 Sample 4-1

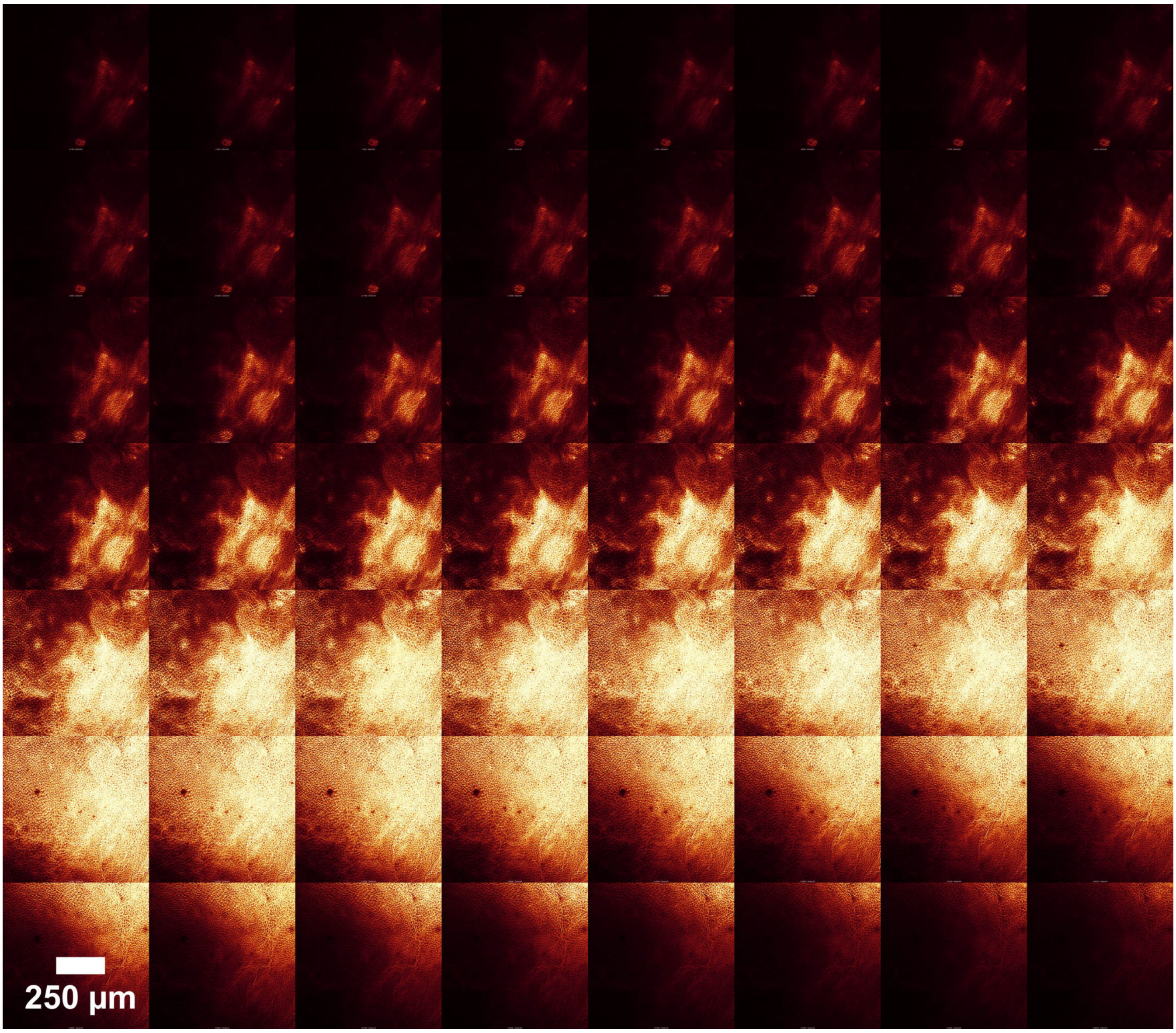

Figure S6. Sample 4-1 Montage. Images are ordered row-wise from left to right. An additional video is attached.

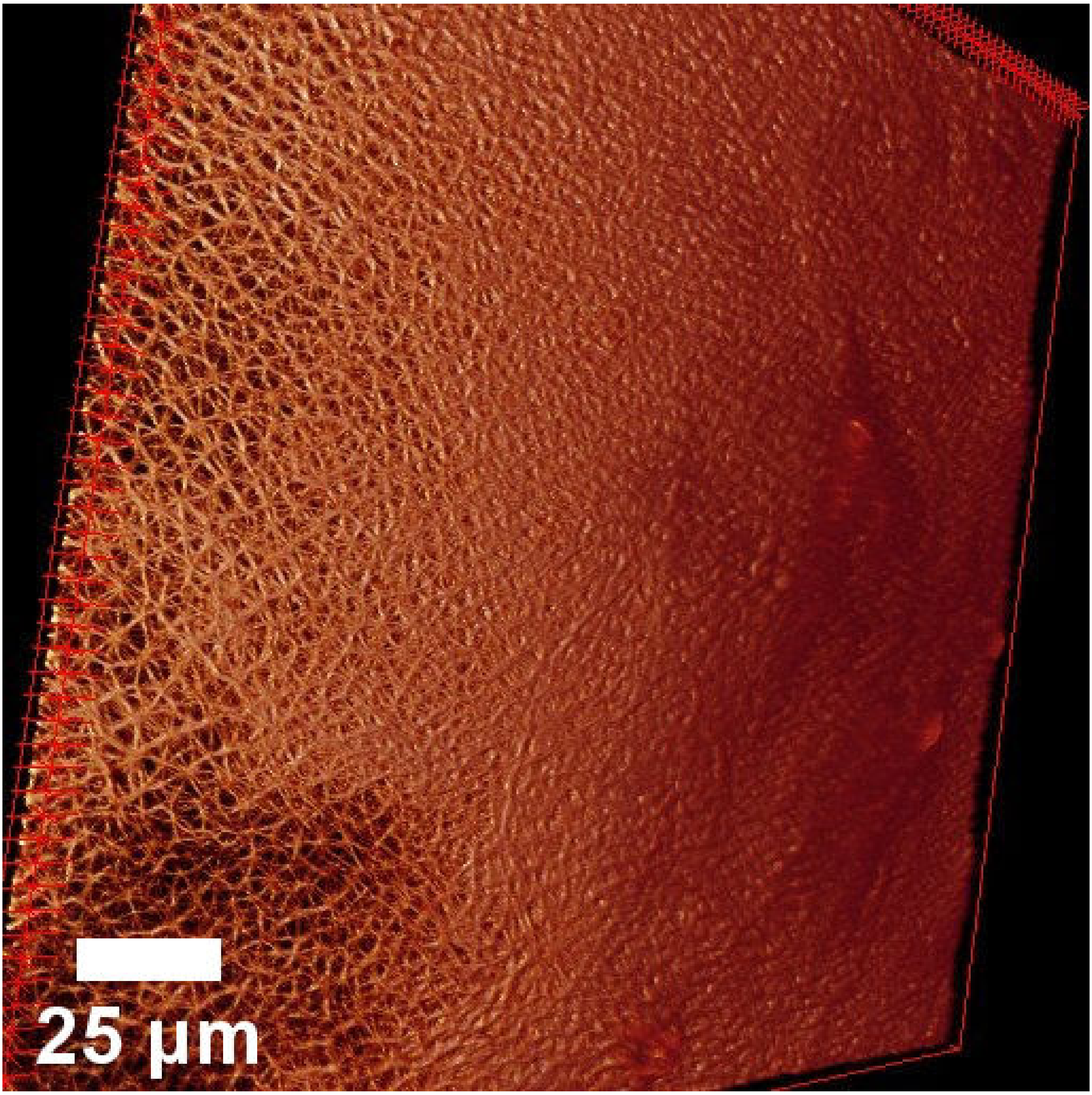

Figure S7. Sample 4-1 3D reconstruction. An additional video is attached.

3.3 Sample 4-2

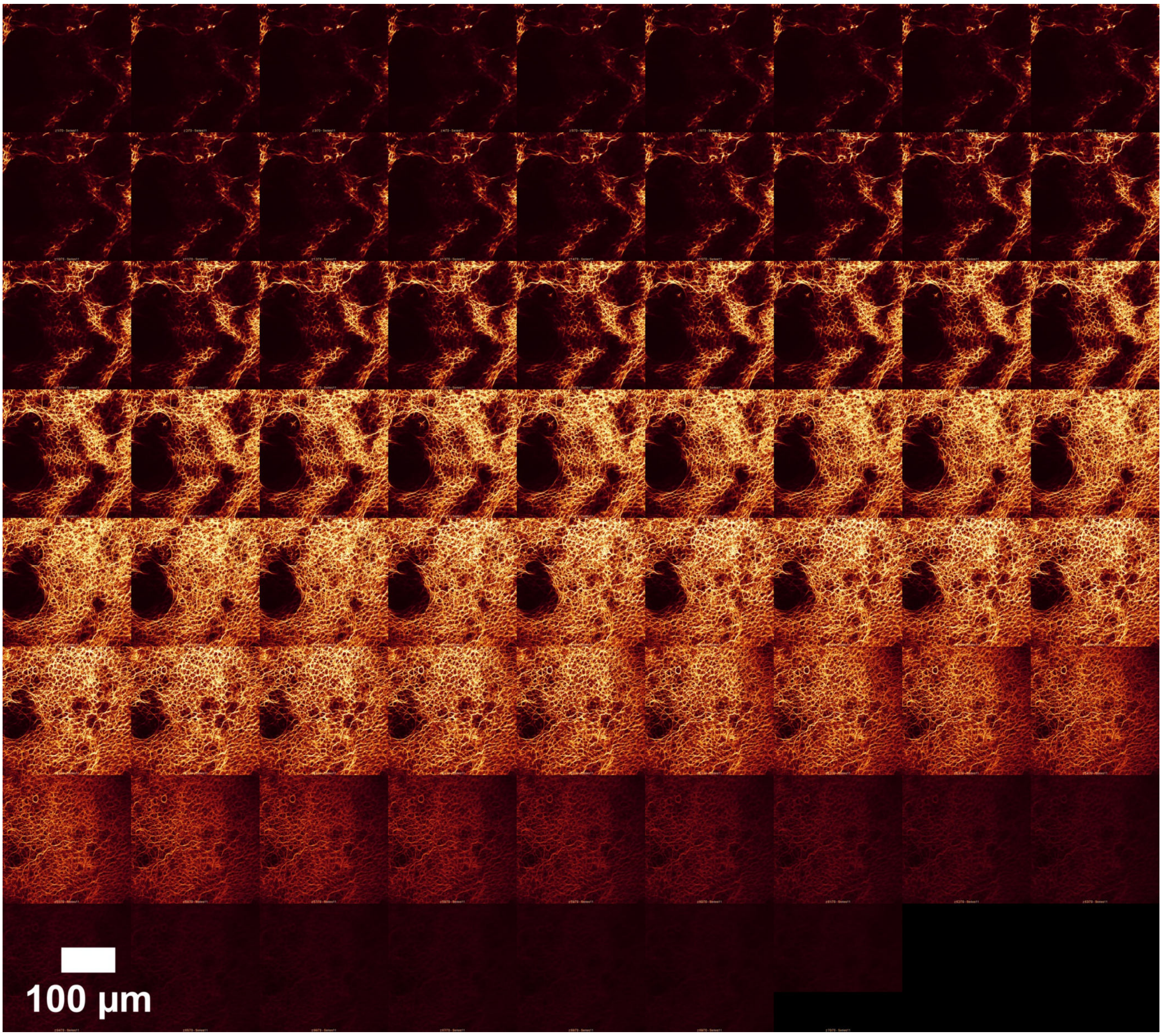

Figure S8. Sample 4-2 Montage. Images are ordered row-wise from left to right. An additional video is attached.

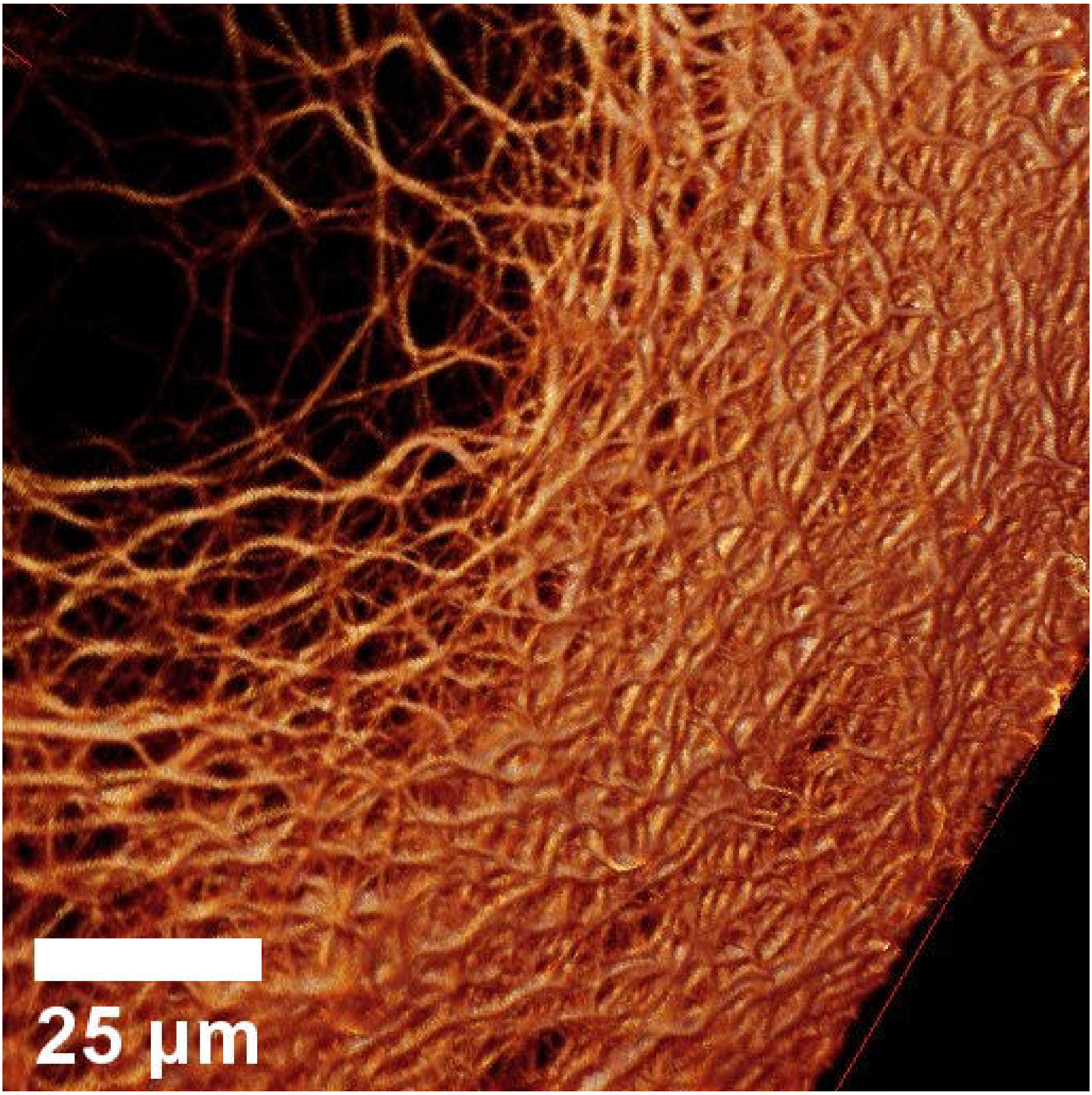

Figure S9. 3D reconstruction of sample 4-2. An additional video is attached.
